# Aminopurvalanol A, a Potent, Selective, and Cell Permeable Inhibitor of Cyclins/Cdk Complexes, Causes the Reduction of *in Vitro* Fertilizing Ability of Boar Spermatozoa, by Negatively Affecting the Capacitation-Dependent Actin Polymerization

**DOI:** 10.3389/fphys.2017.01097

**Published:** 2017-12-22

**Authors:** Nicola Bernabò, Luca Valbonetti, Luana Greco, Giulia Capacchietti, Marina Ramal Sanchez, Paola Palestini, Laura Botto, Mauro Mattioli, Barbara Barboni

**Affiliations:** ^1^Faculty of Bioscience and Technology for Food, Agriculture and Environment, University of Teramo, Teramo, Italy; ^2^School of Medicine and Surgery, University of Milano Bicocca, Monza, Italy; ^3^Istituto Zooprofilattico Sperimentale dell'Abruzzo e del Molise “G. Caporale”, Teramo, Italy

**Keywords:** Aminopurvalanol A, actin, Cyclins/Cdk complexes, sperm capacitation, acrosome reaction, cytoskeleton, cell cycle, *in vitro* fertilization

## Abstract

The adoption of high-througput technologies demonstrated that in mature spermatozoa are present proteins that are thought to be not present or active in sperm cells, such as those involved in control of cell cycle. Here, by using an *in silico* approach based on the application of networks theory, we found that Cyclins/Cdk complexes could play a central role in signal transduction active during capacitation. Then, we tested this hypothesis in the *vitro* model. With this approach, spermatozoa were incubated under capacitating conditions in control conditions (CTRL) or in the presence of Aminopurvalanol A a potent, selective and cell permeable inhibitor of Cyclins/Cdk complexes at different concentrations (2, 10, and 20 μM). We found that this treatment caused dose-dependent inhibition of sperm fertilizing ability. We attribute this event to the loss of acrosome integrity due to the inhibition of physiological capacitation-dependent actin polymerization, rather than to a detrimental effect on membrane lipid remodeling or on other signaling pathways such as tubulin reorganization or MAPKs activation. In our opinion, these data could revamp the knowledge on biochemistry of sperm capacitation and could suggest new perspectives in studying male infertility.

## Introduction

Mammalian spermatozoa, immediately after ejaculation, are virtually infertile and reach their full fertilizing ability only after they reside within the female genital tract for hours to days, depending on the species. This process, called capacitation, implies marked changes in the whole sperm machinery at both membrane and cytosolic levels. Since spermatozoa are transcriptionally silent and their lipid metabolism is very limited (Vazquez and Roldan, 1997a,b), they are unable to synthesize new molecules, adapting their biochemical machinery by modulating the architecture of their membrane (Gadella and Harrison, 2002; Botto et al., 2010; Barboni et al., 2011; Boerke et al., 2013; Gadella and Luna, 2014) and cytoskeleton (Cohen et al., 2004; Daniel et al., 2010; Bernabò et al., 2011). In particular, sperm membrane are divided into several regions known as domains: the apical ridge area, the pre-equatorial area, the equatorial area, the post-equatorial area, the midpiece and the tail. Each of them has a specific chemical composition and is involved in different biological activities (sperm egg interaction, exocytosis of acrosome content, motility, etc…). In turn, each domain contains specialized areas of membranes organized in a liquid ordered phase (L_O_), the lipid microdomains, surrounded by a more fluid liquid disordered (L_D_) membrane. They contain high concentrations of cholesterol, sphingomyelin, gangliosides, phospholipids with saturated long-chain acyl chains, and specific receptors and proteins such as glycosylphosphatidylinisotol (GPI) anchored proteins, caveolin, and flotillin (Botto et al., 2010; Leahy and Gadella, 2017). In addition, as it has been observed in others mammalian cells, the inner and the outer leaflet of membranes have a different chemical composition, with the aminophospholipids phosphatidylserine (PS) and phosphatidylethanolamine (PE) more concentrated in the inner leaflet and the choline phospholipids sphingomyelin (SM) and phosphatidylcholine (PC) more concentrated in the outer leaflet (Gadella and Luna, 2014). During capacitation, membranes undergo a deep rearrangement that affects their composition and their biophysical properties: several lipids displace from one leaflet to the other one, the membrane fluidity increases, and the activity of several enzymes is modulated, due to the activation of specific signaling pathways (Romarowski et al., 2015).

The reorganization of the membranes is functionally and physically linked to the reorganization of actin cytoskeleton (the cytosol of spermatozoa is virtually absent) with the increase in actin polymerization in sperm head region (Breitbart et al., 2005; Bernabò et al., 2011; Ickowicz et al., 2012). Nowadays, we have several information about the biochemistry of capacitation, but many aspects are still unknown.

Recently, the adoption of high-througput technologies is providing new data on virtually all the biological systems studied. In particular, in developmental and reproductive biology the so called—*omics* are extensively employed in studying basic and applied biology of embryo development (Aleksandrova et al., 2016; Garcia-Herrero et al., 2016), ovarian phisiology (Hasegawa et al., 2009; Chronowska, 2014), oocyte maturation (Virant-Klun et al., 2016; Wang et al., 2016), sperm differentiation, and acquisition of fertilizing ability (Amaral et al., 2014; Jodar et al., 2016a,b). With regard to this last issue, several studies have been carried out to study the molecular mechanisms involved in capacitation, providing important information. On one hand, they lead to the identification of several new proteins involved in capacitation and in fertility; on the other hand, they are suggesting the involvement in these processes of proteins that in other cellular models are known to play important roles in pathways related to the cell cycle and its control. Interestingly, in a recent study carried out by reviewing the data referred to proteomic analysis on human spermatozoa, it has been pointed out that cell cycle related proteins were detected as likely active pathways in human sperm. The authors mentioned this finding concluding that “the meiotic proteins might be remnants of spermatogenesis, with no function in mature sperm” (Amaral et al., 2014). Since, in our opinion, it is quite possible that these proteins could express different or new activities in the context of male gametes, we carried out an *in silico* and *in vitro* experiment. First, using a computational modeling approach, we identified the proteins that exert the stronger control in cell cycle as target for the *in vitro* experiments. Then, we tested the effects of a potent and specific inhibitor of the identified proteins on *in vitro* capacitation and IVF experiments.

## Materials and methods

### *In silico* experiment

#### Networks realization, analysis, and visualization

**Figure 1:**
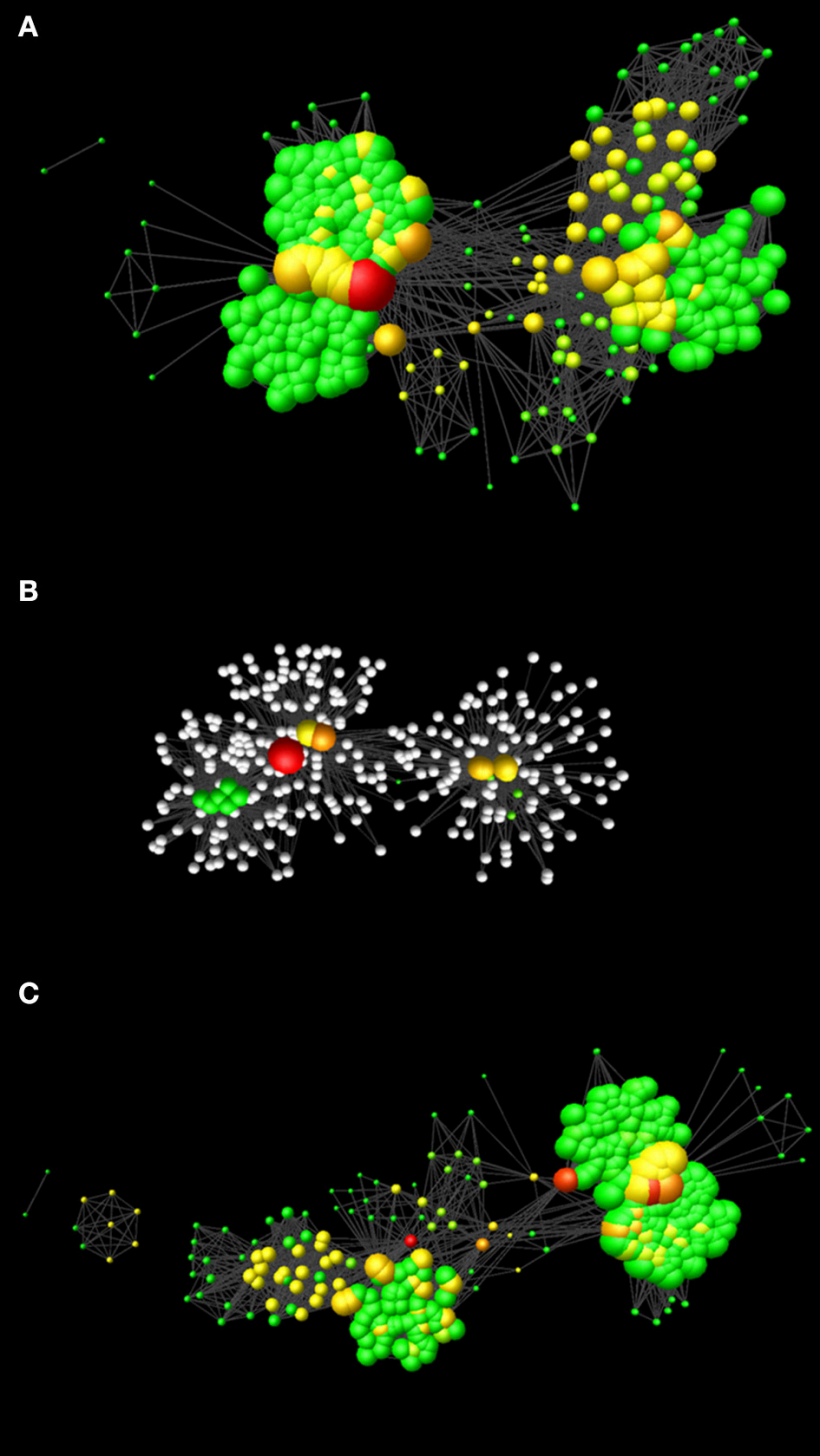
Network models realized in this study. **(A)** Network representing the molecular events involved in cell cycle and cell cycle control (CCN). **(B)** Network representing the nodes affected by Aminopurvalanol A and those under their direct control. **(C)** Network resulting after the removal of the nodes affected by Aminopurvalanol A from CCN. In all the cases, the networks are represented with the Perfuse Force Directed Layout, and the node diameter is proportional to the node degree and the node color depends on the betweenness centrality (green = lower values, red = higher values).

We performed a network representing the molecules involved in cell cycle and its control. As data source, we used Reactome (http://www.reactome.org/), a free, open-source, curated and peer reviewed pathway database (Cheng et al., 2015). We downloaded the data referred to the pathway “Cell cycle, mitotic” (Stable Identifier R-SSC-69278.1) filtered for species Sus scrofa and we used them to generate a network representing the molecules involved in cell cycle and its control (Cell Cycle Network, CCN, see Figure 1A), by using Cytoscape 3.3.0. As network view renders, we used Cy3D (http://apps.cytoscape.org/apps/cy3d), using a 3D force-directed layout. All the calculation were computed with Network Analyzer version 2.7 (http://apps.cytoscape.org/apps/networkanalyzer). We assessed the following topological parameters:

***Connected components***: The number of networks in which any two vertices are connected to each other by links, and which is connected to no additional vertices in the network.
***Number of nodes***: The total number of molecules involved.
***Number of edges***: The total number of interactions found.
***Clustering coefficient***: It is calculated as *C*I = 2*n*I/*k*I(*k*I−1), where *n*I is the number of links connecting the *k*I neighbors of node I to each other. It is a measure of how the nodes tend to form clusters.
***Network diameter***: The longest of all the calculated shortest paths in a network.
***Shortest paths***: The length of the shortest path between two nodes *n* and *m* is *L*(*n*,*m*). The **s**hortest path length distribution gives the number of node pairs (*n*,*m*) with *L*(*n*,*m*) = *k* for *k* = *1,2*,…
***Characteristic path length***: The expected distance between two connected nodes.
***Averaged number of neighbors***: The mean number of connections of each node.
***Node degree***: The number of interaction of each node.
***Node degree distribution***: It represents the probability that a selected node has *k* links.
**γ:** The exponent of node degree equation.
***R***^2^: The coefficient of determination of node degree vs. number of nodes, on logarithmized data.

To predict the effects of Aminopurvalanol A on network topology, in keeping with the literature indications, we removed from CCN the following records: CDK1/CCNB; CDK2/CCNA; CDK5/p25; CDK4/CCND).

#### Cluster analysis on CCN topological parameters

The nodes of CCN were subjected to a multivariate cluster analysis based on their node degree, betweenness centrality, closeness centrality, and stress centrality.

In particular, the betweenness centrality was computed as:

$$\begin{array}{l} C_{b}\left(n\right)=\sum_{s\neq n\neq t}\left(\sigma_{st}\left(n\right)/\sigma_{st}\right), \end{array}$$

where *s* and *t* are nodes in the network different from *n*, σ_*st*_ denotes the number of shortest paths from *s* to *t*, and σ_*st*_ (*n*) is the number of shortest paths from *s* to *t* that *n* lies on. The betweenness centrality value of each node *n* is normalized by dividing by the number of node pairs excluding *n*: (*N*-1)(*N*-2)*/2*, where *N* is the total number of nodes in the connected component that *n* belongs to, thus it ranges between 0 and 1. This measure is related to the amount of control that a node exerts within the network.

The closeness centrality is defined as the reciprocal of the average shortest path length and is computed as follows:

$$\begin{array}{l} C_{c}\left(n\right)=1/avg\left(L\left(n,m\right)\right), \end{array}$$

where *L*(*n*,*m*) is the length of the shortest path between two nodes *n* and *m*. It ranges between 0 and 1 and it is a measure of how fast information spreads from a given node to other reachable nodes.

The stress centrality of a node *n* is the number of shortest paths passing through *n*. A node has a high stress if it is traversed by a high number of shortest paths.

The cluster analysis was performed using a paired group algorithm (UPGMA) with a Euclidean similarity index (Past3).

#### Identification of bottleneck nodes within HSIN 3.1

The Bottlenecks analysis was carried out by a Cytoscape plugin CytoHubba. CytoHubba implements the following algorithm for bottleneck calculation: let Ts be a shortest path tree rooted at node s.

$$\begin{array}{l} \text{BN}\left(\text{v}\right)=\Sigma \text{s}\epsilon \text{Vps}\left(\text{v}\right) \end{array}$$

where ps(v) = 1 if more than |V(Ts)|/ 4 paths from node s to other nodes in Ts meet at the vertex v; otherwise ps(v) = 0 (Cheng et al., 2015).

### *In vitro* experiment

#### Chemicals

If not otherwise indicated, all the chemicals we purchased from Sigma Aldrich (Darmstadt, Germany) and were of the purest analytical grade. Primary antibodies against Phospho-p44/42 MAPK (Erk1/2) (Thr202/Tyr204) and p44/42 MAPK (Erk1/2) were from Cell Signaling (Danvers, MA). Secondary antibodies for enhanced chemiluminescence (ECL) detection were anti-rabbit HRP conjugates from Pierce (Rockford, IL). All material for electrophoresis was from Bio-Rad (Milan, Italy).

#### Spermatozoa preparation

Semen samples were purchased at Inseme s.p.a. (Modena, Italy) and processed by using an already validated protocol (Maccarrone et al., 2005; Bernabò et al., 2010b; Barboni et al., 2011). Briefly, the spermatozoa were washed twice in Dulbecco's Phosphate-Buffered Saline (DPBS) at 1,500 g for 10 min. Then they were resuspended in TCM199 containing 26.1 mM sodium bicarbonate added with 13.9 mM glucose, 1.25 mM sodium pyruvate, 2.25 mM calcium lactate, and 1% of Penicillin-Streptomycin solution (containing 10,000 U/ml penicillin and 10,000 U/ml streptomycin/ml) (300 mOsm/kg, pH 7.4) as capacitation medium. The incubation under capacitating condition was carried out at the final concentration of 1 × 10^8^ cells/ml for 4 h at 38.5°C in 5% CO_2_ humidified atmosphere (Heraeus, Hera Cell). Only the samples showing a mean viability, assessed as previously described (Bernabò et al., 2007), of at least 90% at the beginning of the culture were considered for the following analysis. The sperm samples were processed as T0, incubated under control conditions (CTRL) or were constantly maintained in the presence of 2, 10, or 20 μM of Aminopurvalanol A for 4 h.

#### Monitoring of the effect of aminopurvalanol a on acrosome integrity

The acrosome integrity was monitored by using *Pisum sativum* agglutinin (PSA) staining. For this purpose we used a two staining technique with Hoechst 33258 and FITC-PSA, able to identify live unreacted and reacted spermatozoa (Mattioli et al., 1996; Bernabò et al., 2007). For each CTRL and treated sample at least 100 cells have been assessed under fluorescence microscope. The experiments have been repeated at least three times in independent experimental set-ups and using semen samples from different animals.

#### IVF experiments

To verify the effects of Aminopurvalanol A treatments on spermatozoa fertilizing ability, an *in vitro* fertilization (IVF) assay was carried out, using a validated protocol (Bernabò et al., 2007). The ovaries of prepubertal gilts were collected at a local slaughterhouse and transported to the laboratory within 1 h, at 25°C. After washing in normal saline, the ovaries were mechanically dissected under sterile conditions in Dulbecco's phosphate buffer with 0.4% BSA. Follicles of 4–5 mm diameter, were selected on the basis of their translucent appearance, good vascularization, and compactness of their granulosa layer and cumulus mass. Healthy follicles were opened and the oocytes were recovered and cultured to MII stage in four wells dishes containing 500 μl TCM 199 added with 10% FCS, 70 mg/l kanamicin, ITS 10 ml/ml and 1 mg/ml porcine LH and FSH, and follicle walls reversed and placed on a stainless grid to avoid contact with the Petri dish bottom. After 44 h of culture, the oocytes were partially denuded in Hepes-TCM 199 with hyaluronidase (1%) on a warmed stage at 38.5°C under a stereomicroscope. Only oocytes presenting the first polar body (MII stage) under the stereomicroscope were utilized for the IVF assay. IVF was performed in fertilization medium, according to previously validated protocol (Bernabò et al., 2010c, 2015b) by using *in vitro* capacitated control and Aminopurvalanol A treated spermatozoa. The oocytes were coincubated with the spermatozoa at a final concentration of 0.5 × 10^7^ cells/ml, for 1 h, then were gently removed from the Petri dish, transferred to fresh medium and maintained in culture for at least 12 h. IVF results have been expressed as fertilization rate (% of penetrated oocytes), incidence of polyspermy (% of polyspermic oocytes), and number of penetrating spermatozoa/polyspermic oocyte (Abeydeera and Day, 1997; Bernabò et al., 2007). We carried out three independent experiments, each of them with total 148 oocytes, for a total of 444 oocytes observed.

#### Phalloidin staining

In order to assessing the effect of Aminopurvalanol A treatment on actin polymerization, the sperm samples were fixed in absolute ethanol for at least 1 h at −20°C and spread on microscope slides. After air-drying, they were incubated with FITC-conjugated phalloidin (3 μM in PBS) for 60 min, washed two times with distilled water and mounted with Vectashield mounting medium (Vector H-1000). For confocal microscopy set up refer to next paragraph.

#### Triple staining for acrosome, actin, and nucleus

Sperm samples were fixed in absolute ethanol for at least 1 h at −20°C, spread on glass slides and air-dried. Next, they were incubated with TRITC-phalloidin 80 nM in PBS for 50 min followed by incubation with DAPI 0.2 mM in PBS for 15 min and then FITC-conjugated PSA at 50 μg/ml in PBS for 20 min. Finally, they were washed three times with tap water and mounted with Vectashield mounting medium (Vector H-1000).

The acquisition of triple staining was realized with Nikon A1r laser confocal scanning microscope, equipped with a Plan Apo λ 100X Oil objective, detector Galvano, with a pinhole size of 69 μM and a pixel size 0.04 um. We used an averaged 2 mode in channels series as follows:

Channel 1: FITC: λ_exc_ = 488 nm; λ_em_ = 525/50 nm, at 6.6% of the maximum laser power
Channel 2: DAPI: λ_exc_ = 404 nm; λ_em_ = 450/50 nm at 3% of the maximum laser power
Channel 3: TRITC: λ_exc_ = 561.5 nm; λ_em_ = 595/50 nm at 1.8% of the maximum laser power

#### Tubulin immunocytochemistry

Sperm samples were fixed in absolute ethanol for at least 4 h at −20°C, spread on glass slides, air-dried, and washed with phosphate buffer solution (PBS). They were permeabilized with 0.5% Triton X-100 in PBS for 5 min and washed 3 times at 5 min intervals with PBS. After sperm were immersed in 5% bovine serum albumin (BSA) in PBS for 30 min to block non-specific sites and incubated overnight at 4°C with monoclonal anti-αtubulin antibody produced in mouse (Sigma Aldrich) diluted 1:500 in PBS containing 1% BSA. Next, the slides were washed three times at 5 min intervals with PBS. The bound antibody was detected using anti-mouse cy3 TRICT-conjugated (Sigma Aldrich) diluted 1:500 in PBS containing 1% BSA incubated for 1 h at room temperature and followed by washing three times at 5 min intervals with PBS. Finally, the slides were mounted in Vectashield mounting medium (Vector H-1000). Sperm samples incubated with PBS without the primary antibody were used as negative controls.

#### Confocal analysis of spermatozoa-oocyte binding

To assess the acrosome status of spermatozoa after the interaction with oocytes, we fixed in PBS with 4% paraformaldehyde the oocytes incubated with spermatozoa under IVF conditions for 30 min at 4°C. Then, after three washes in PBS, the oocytes were permabilized in PBS with 0.1% Triton X100, washed in PBS and stained with Phalloidin and PSA (same conditions as above). After that, the oocytes were mounted and observed with Nikon A1r laser confocal scanning microscope, equipped with a Plan Apo λ 60X Oil objective (numerical aperture: 1.4; Refreactive Index: 1.515). For scanning we used the specific function Nikon A1 Piezo Z Drive, detector Galvano, pinhole size of 39.6 μm, Z-step: 0.15 μm, and 572 slices, in channel series, as follows:

Channel 1: DAPI: λ_exc_ = 404 nm; λ_em_ = 450/50 nm at 7.6% of the maximum laser power
Channel 2: FITC: λ_exc_ = 488 nm; λ_em_ = 525/50 nm, at 3.1% of the maximum laser power
Channel 3: TRITC: λ_exc_ = 561.5 nm; λ_em_ = 595/50 nm at 3.1% of the maximum laser power

#### Evaluation of aminopurvalanol a effect on spermatozoa membrane fluidity by fluorescence recovery after photobleaching (FRAP)

After washing and incubating with capacitation medium, spermatozoa were stained with the lipophilic fluorescent molecule DilC12(3) perchlorate (ENZ-52206, Enzo Life Sciences, USA) used in a dilution 1:1,000. Incubation was carried out for 15 min at 38.5°C in 5% CO_2_ humidified atmosphere (Heraeus, Hera Cell). Spermatozoa were then washed twice with PBS and centrifuged for 10 min at 850 g. The protocol was performed at T0 and after 2 h of incubation under capacitating conditions, with control and treated spermatozoa (10 and 20 μM Aminopurvalanol A). We used these experimental conditions because they are referred to the onset of acrosome damage. FRAP experiments were performed with the confocal microscope Nikon A1r laser confocal scanning microscope equipped with the NIS-Element software, using a Plan Apo λ 100X Oil objective (numerical aperture: 1.45; zoom: 1X; Refreactive Index: 1.515; pinhole size: 69 μm; 1 picture every 0.512 s). Fluorescence bleaching and recovery were conducted as follows: λ_exc_ = 561.5 nm; λ_em_ = 595/50 nm with 1 scan for basal fluorescence record at 2.4% of the maximum laser power, 1 scan at 100% laser power for bleaching, and 25 scans for monitoring recovery at 2.4% of the maximum laser power. Recovery curves were recorded and analyzed by using the simFRAP plug-in for Fiji ImageJ (https://imagej.nih.gov/ij/plugins/sim-frap/index.html) (Blumenthal et al., 2015). It computes the diffusion coefficients of fluorescent dye embedded in cell membrane, regardless of bleaching geometry. The algorithm is based on fitting a computer-simulated recovery to actual recovery data of a FRAP series. We set the requested parameters as following: pixel size: 0.12 μm; acquisition time per frame: 0.12 s. the results were expressed as diffusion coefficient (cm^2^/s). We carried out three independent experiments, performed on different boars and on different days.

#### Western blotting

Sperm samples were incubated as previously described either under CTRL or treatment conditions. After 5, 10, 15, 30, 60, 120, and 180 min the spermatozoa were washed twice in PBS, counted and then frozen at −80°C. The homogenates (Hom) were prepared according to Hyne and Garbers (1979). Briefly, the sperm pellet was resuspended in a hypotonic buffer (2 mM Tris [pH 7.2], 12 mM NaCl) with protease inhibitors (10 μg/ml aprotinin, 10 μg/ml leupeptin, 1 mM PMSF) and since phosphorylated proteins were investigated, phosphatases inhibitors were added (Yanagida et al., 2000). The final sperm concentration in the hypotonic buffer was 0.5 × 10^8^ spermatozoa/ml. The cells were homogenized with 20 strokes in a tight fitting glass Douce homogenizer on ice and the protein content was quantified by a micro-BCA assay from Sigma. Thereafter, 20 μg of Hom were loaded on SDS-PAGE (8% polyacrylamide gel) and submitted to electrophoresis. Subsequently, proteins were transferred to membranes that were stained with Ponceau S to assess proper transfer. Blots were washed with TBS and subsequently blocked for 1 h in TBS-T/BSA. After blocking, blots were incubated overnight with the primary antibody diluted in TBS-T/BSA [anti-Phospho-p44/42 MAPK (Erk1/2) 1:1,000, anti-p44/42 MAPK (Erk1/2) 1:1,000], and then for 1.5 h with HRP-conjugated anti-rabbit IgG (10,000-fold diluted in TBS-T/BSA). Homogenates were obtained from three independent experiments. Proteins were detected by ECL with the Super Signal detection kit (Pierce). Immunoblot bands were analyzed by ImageQuant™ TL (GE Healthcare Life Sciences), program 1D gel analysis.

### Data analysis

#### Boltzmann sigmodal model

The loss of acrosome integrity was evaluated in a time-concentration assay, by using a Boltzmann sigmodal model that allows calculating the values of bottom, top and 50% effect (E50), i.e., the time at which the effect of the analyses variable was at the 50% of maximum. As measure of the goodness of fitting, we used the determination coefficient (*R*^2^). All the calculation have been done with Graphpad Prism.

#### Kernel density estimation analysis

To identify the sub-populations in sperm samples stained with FITC-PSA we used a Kernel Density Estimation (KDE) technique. It is a non-parametric analysis able to estimate the probability density function of a variable, thus allowing to infer if it is constituted by subpopulations of data (Bernabò et al., 2015a). The analysis was carried out by using Past3.

#### Statistical analyses

The data were checked for normal distribution (D'Agostino and Pearson normality test), then they were compared by parametric or non-parametric tests, as reported in results section and in figure legends (Graphpad 5). Data reported in this paper are referred to at least three independent experiments, each performed in duplicate and reported as mean ± standard deviation or median and 25° and 75° percentile. The differences were considered significant and highly significant for *p*-values of <0.05 and <0.01, respectively.

## Results

### *In silico* experiments on network topology

The network representing the molecules involved in cell cycle and its control, CCN, was composed by two connected components: one of 2 nodes and 1 edge, and the other main component (CCN main component CCN_MC) of 331 nodes and 10,419 edges (Figure 1A), their main topological parameters are listed in Table 1.

**Table 1:** Results of networks topological analysis.

**Topological parameter**	**CCN_MC**	**CCN_MC_AA**
Connected components	1	2
Number of nodes	331	320
Number of edges	10419	9476
Clustering coefficient	0.880	0.905
Network diameter	5	6
Characteristic path length	2.397	2.665
Averaged number of neighbors	62.577	59.225
Shortest paths	109232 (98%)	95848 (93%)

To identify the most influencing node/s within the network, we analyzed different topological parameters. In particular, we identified the hubs, finding that the most linked node was CDK1 (which represents the cdk1 protein). Then, we performed a multivariate cluster analysis, based on the topological parameters related to the control of network (node degree, betweenness centrality, closeness centrality, stress centrality). Interestingly, it confirmed the specificity of the CDK1 topology (see Supplementary Information 1).

Lastly, we identified the bottleneck nodes within the network. The nodes showing the higher score are CDK1 (score 143.0), CCNB1 (score 108.0), and CCNA2 (score 73.0).

To simulate the effect of using a specific inhibitor of cdk1 function (Aminopurvalanol A), we performed a computational experiment by removing from the network cdk1 and the related nodes (see discussion): cdk2, cdk4, cdk 5, cyclin A, cyclin B, cyclin D, and p25 (Figure 1B). The results are shown in Table 1 and Figure 1C. As it is evident, this simulation suggests that the treatment with Aminopurvalanol A significantly changes the global topology of the network.

### *In vitro* experiments on sperm function

#### Effect of aminopurvalanol a on acrosome integrity

**Figure 2:**
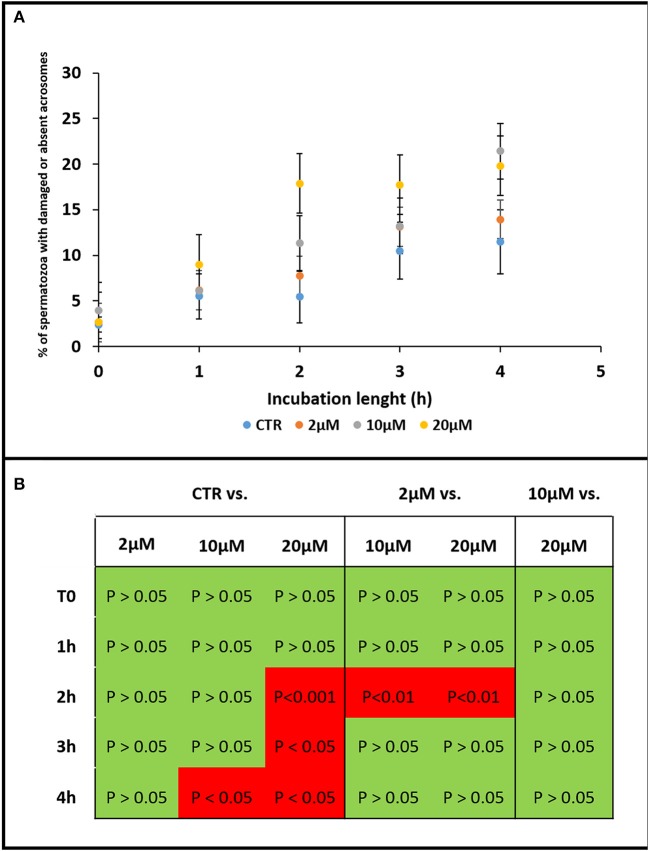
Graph showing the effect of Aminopurvalanol A on acrosome integrity. The effects of Aminopurvalanol A at different concentration and incubation times on the percentage of spermatozoa with absent or damaged acrosome were shown **(A)**. The statistically relevant differences are reported **(B)**. The data are reported as mean±standard deviation, and the comparisons were carried out with ANOVA two ways, followed by Tukey's *post-hoc* test.

First, we found that at concentration of Aminopurvalanol A higher than 20 μM the sperm viability and gross motility were negatively affected by the treatment. For this reason, we assessed the effects of Aminopurvalanol A on the acrosome integrity on sperm samples incubated under capacitating conditions and exposed to concentration of 2, 10, and 20 μM. As a result, we found that the different concentrations of Aminopurvalanol A exert a significant effect on the percentage of spermatozoa with damaged or absent acrosome (15.74% of total variation, *p* < 0.0001), depending on the length of incubation (56.76% of total variation, *p* < 0.0001), while the interaction of treatments with the incubation time had not statistically evident effects (9.66% of total variation, *p* = 0.1188) (Figure 2).

The kinetics of the effect exerted by Aminopurvalanol A on sperm acrosome integrity is different among the treatments: from the result of a time-effect curve, it is evident that the E50 (i.e., the length of incubation in which the 50% of sperm acrosome damage is reached), is similar in CTRL, 2 and 10 μM, ranging from 2.1 to 2.9 h, while it is less than one-half in 20 μM (about 1.1 h). The maximum values are similar for 10 and 20 μM.

### Actin polymerization kinetics

**Figure 3:**
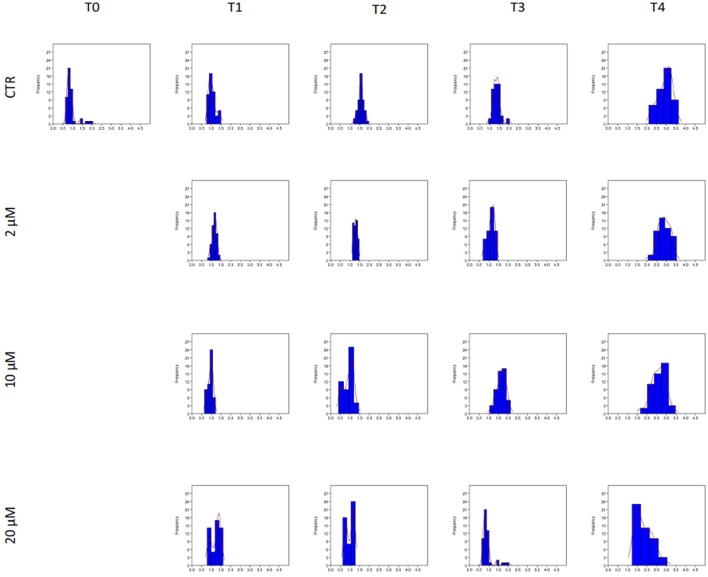
Effect of Aminopurvalanol A on actin polymerization. The histograms represent the subpopulation of spermatozoa individuated by Kernel Density Estimation. On x axis are reported the values of fluorescence emitted by spermatozoa stained with FITC-conjugated falloidin, expressed as arbitrary units.

As it is shown in Figure 3, the amount of F-actin, expressed as Arbitrary Fluorescence Units emitted by FITC-conjugated phalloidin, is increasing during the incubation of spermatozoa under condition able to promote the capacitation (CTRL). Interesting, it is negatively affected by Aminopurvalanol A treatment, in a dose dependent manner. In particular, in 10 and 20 μM Aminopurvalanol A treated samples the sub-populations identified with KDE show a pattern more similar to that of T0, with a lower number of sperm cells displaying high fluorescence.

### Confocal analysis of actin subcellular localization

**Figure 4:**
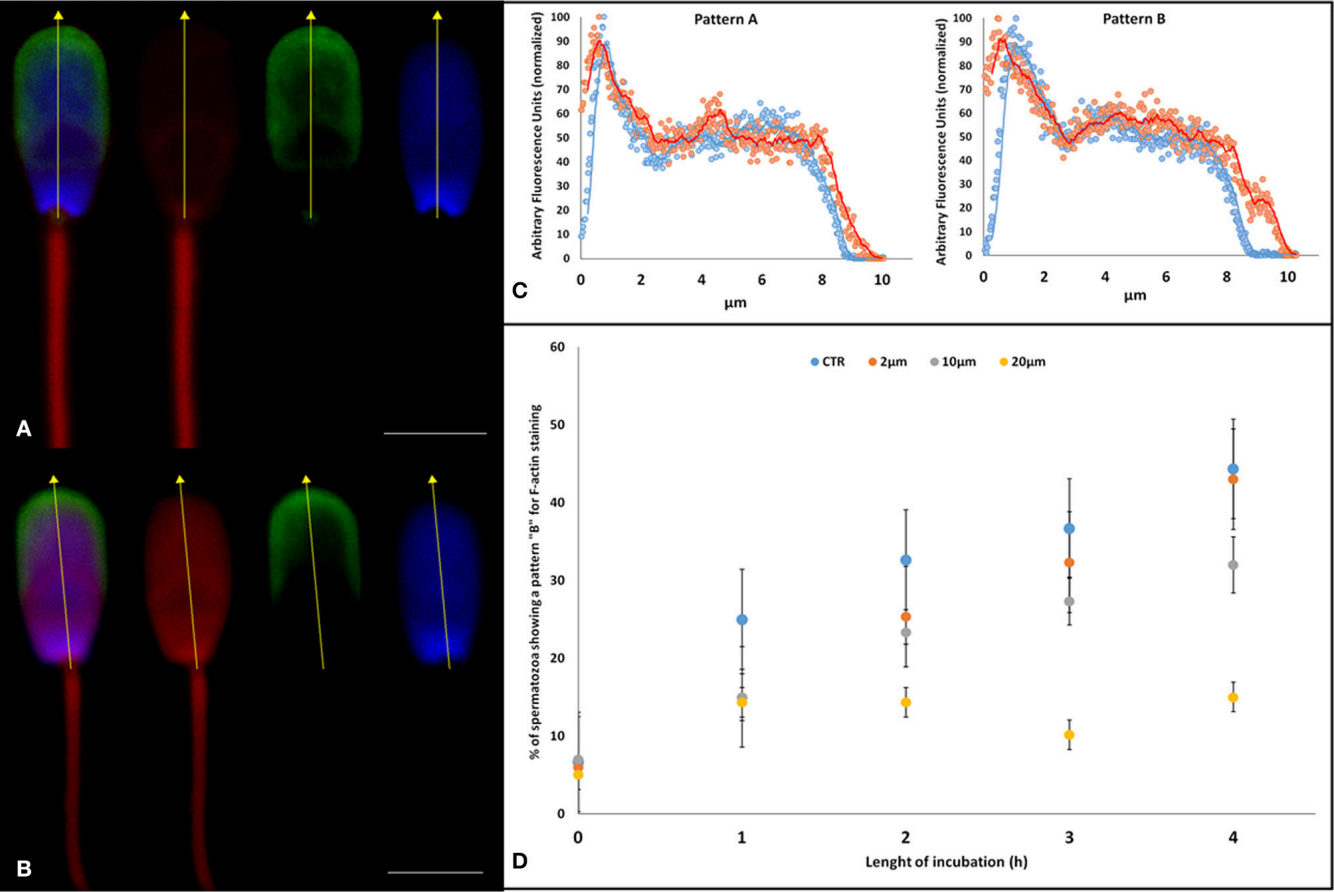
Effect of Aminopurvalanol A on actin subcellular localization. **(A,B)** confocal scanning microscope images of a spermatozoon stained with DAPI (blue, nucleus), TRITC-conjugated falloidin (red, F-actin), and FITC-conjugated PSA (green, acrosome). With this staining technique we identified the acrosome-intact spermatozoa and we assessed the levels of fluorescence emitted by falloidin to astudy the effect of Aminopurvalanol A on actin polymerization using as a reference the nucleus. The fluorescence quantification was carried out along the longitudinal axis of sperm head (yellow dot line). In particular the spermatozoon in **(A)** display a Pattern A, that in **(B)** a pattern B. **(C)** The graphs display the graph resulting from the analysis of fluorescence emission pattern of spermatozoa showing a faint fluorescence emission over post-acrosomal region (Pattern A) and of spermatozoa, displaying high fluorescence emission over anterior area of sperm head (Pattern B). **(D)** Effect of incubation with Aminopurvalanol A, at different time, the percentage of spermatozoa showing Pattern B. The data are represented as mean ± satndard deviation, and the comparison were carried out by ANOVA two ways followed by Tukey's *post-hoc* test. See Table 2 for the results of statistical analysis.

To detail the cellular topology of actin polymerization we used confocal microscopy because by this way, it was possible to observe the specific localization of intracellular fluorescence. We found that, in agreement with our previous work (Bernabò et al., 2010a,b), it was possible to recognize two different patterns of F-actin localization: pattern A, with low florescence emission in the anterior part of the head, and pattern B, with a higher fluorescence emission in the anterior area of spermatozoa head (see Figure 4 left). During the incubation under control conditions, the pattern B significantly increased. Aminopurvalanol A-treated spermatozoa display a dose-dependent reduction of this parameter (Figure 4 right panel), see Table 2.

**Table 2:** Results of the statistical analysis on the effect of different concentrations of Aminopurvalanol on actin polymerization (Pattern B).

**Variability Source**	**p**			
Treatment	**1.21E-15**			
Time	**1.20E-12**			
Interaction	**1.68E-05**			
	**CTRL**	**2 μm**	**10 μm**	**20 μm**
**T1**				
CTRL	1	**0.03477**	**0.03477**	**0.02163**
2 μm		1	1	0.9847
10 μm			1	0.9847
20 μm				1
**T2**				
CTRL	1	**0.04963**	**0.015**	**0.000386**
2 μm		1	0.8176	**0.005939**
10 μm			1	**0.01821**
20 μm				1
**T3**				
CTRL	1	0.3563	**0.02215**	**0.000254**
2 μm		1	0.2534	**0.000391**
10 μm			1	**0.001675**
20 μm				1
**T4**				
CTRL	1	0.9615	**0.009487**	**0.000241**
2 μm		1	**0.01773**	**0.000247**
10 μm			1	**0.001441**
20 μm				1

*The data have been analyzed by ANOVA two-ways followed by Tukey's post-hoc test. In bold are reported statistically significative differences (p < 0.05)*.

### Effect of aminopurvalanol a on tubulin immunocytochemical pattern

**Figure 5:**
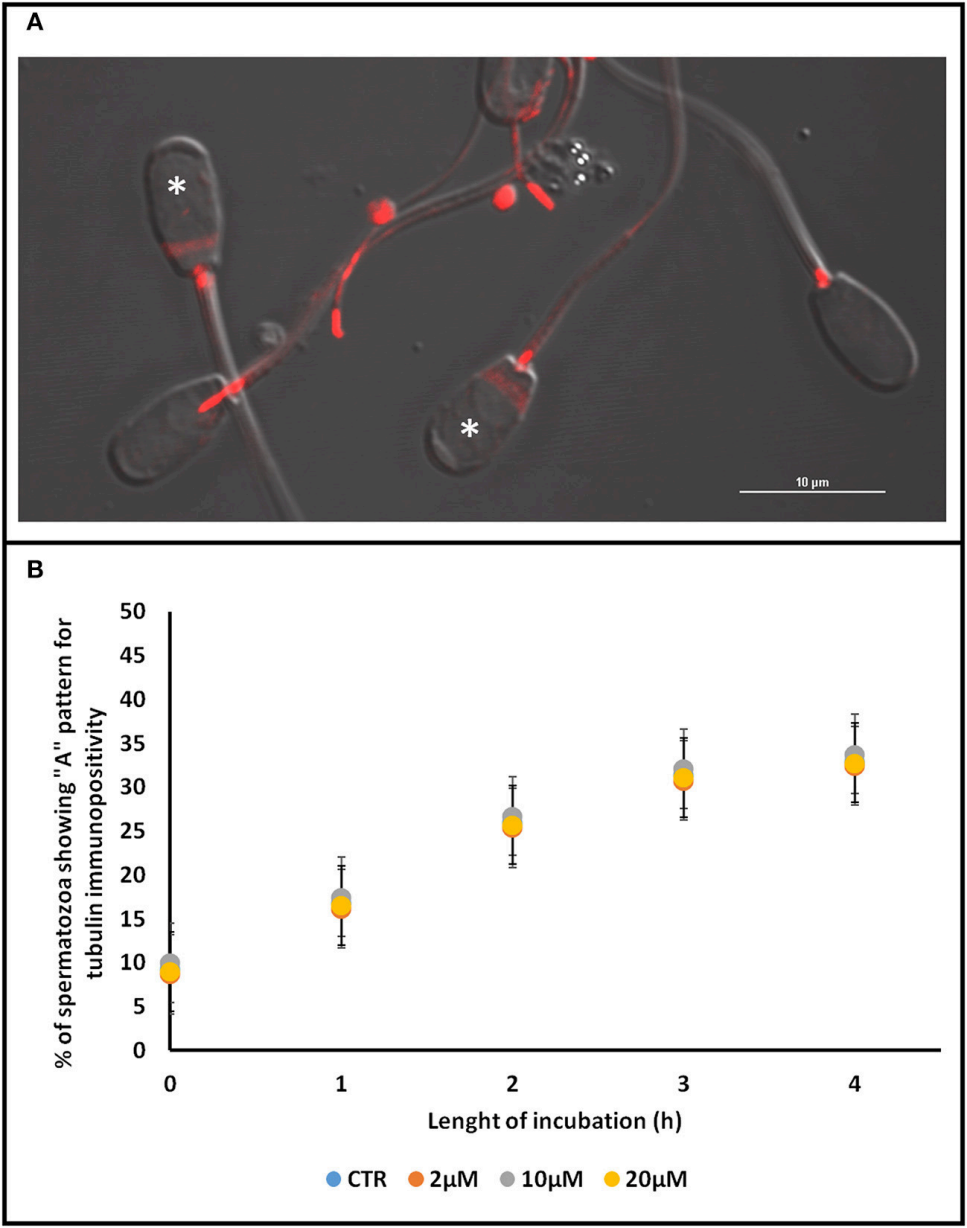
Effect of Aminopurvalanol A on tubulin subcellular localization. In **(A)** are shown the two different patterns of tubulin immunopositivity localization found. In **(B)** the percentage of spermatozoa displaying pattern B, at different incubation times, are reported. For each time point no significative differences were found. The data are represented as mean±satndard deviation, and the comparison were carried out by ANOVA two ways followed by Tukey's *post-hoc* test. See Table 2 for the results of statistical analysis. ^*^Denotes spermatozoa displaying Pattern A.

We identified two different pattern of immunolocalization of tubulin at T0: the pattern A, characterized by immunopositivity at the level of midpiece and at the equator and the pattern B, characterized by an immunopositivity area at the level of midpiece (see Figure 5A).

As it is shown in Figure 5B, during the incubation under control condition the percentage of spermatozoa displaying the pattern A gradually increased from about 10% to about 30%. In any case, the treatment of sperm samples with Aminopurvalanol A did not seem to exert any detectable effect on this parameter (*p* > 0.05), allowing us to infer that this drug do not affect the dynamics of tubulin cytoskeleton.

### FRAP analysis of the effect of aminopurvalanol a treatment on membrane fluidity

**Figure 6:**
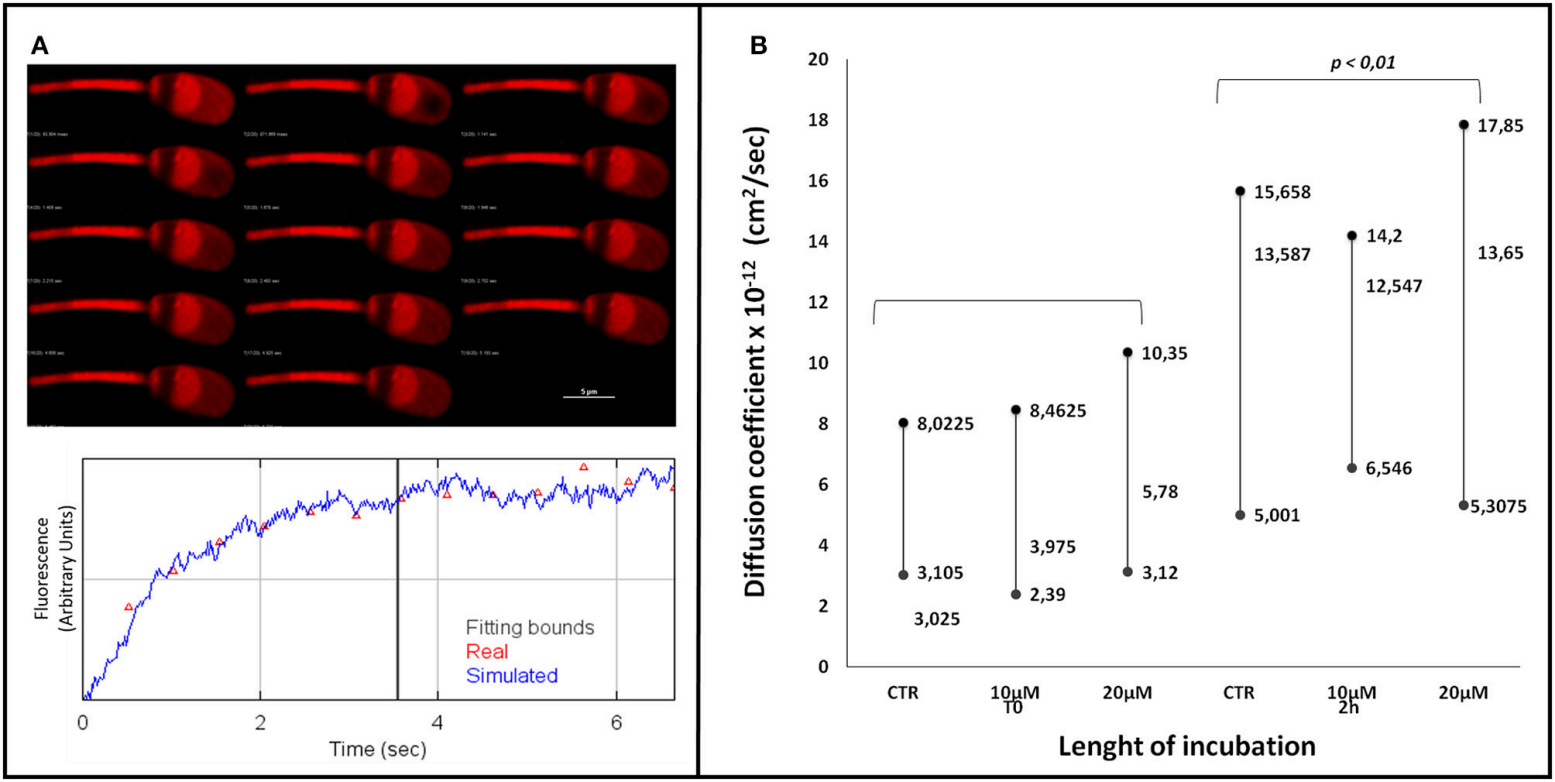
FRAP analysis of spermatozoa stained with DilC12 incubated with or without Aminopurvalanol A. **(A)** gallery showing the FRAP analysis method and the measurement of fluorescence recovery by confocal microscopy. **(B)** Effect of different times of incubation and Aminopurvalanol A concentrations on DilC12 diffusion coefficient. The data are reported as median and 25° and 75° percentile. We found that the data at 2 h incubation were significantly different (*p* < 0.01) from those at T0, while the different treatments did not exerted any significative effect (*p* > 0.05). The comparison were carried out with Kruskall-Wallys test.

The data obtained with FRAP experiment show that under capacitating condition, as expected, the diffusion coefficient of DilC12 increases, likely due to the increased membrane fluidity (Figure 6). The treatment with AP did not change this parameter at T0 neither after 2 h of incubation (*p* > 0.05), thus allowing to suppose that AP did not affected the physico-chemical properties of sperm membranes.

### Effects of aminopurvalanol a treatment on IVF outcome and on sperm-oocyte binding

Based on the results related to the effect of Aminopurvalanol A on acrosome integrity, we decided to carry out an IVF experiment. Under CTRL conditions 67.1 ± 10.4% of oocytes were fertilized, with 85.1 ± 9.5% of polyspermic oocytes and 4.3 spermatozoa/polyspermic oocyte. The treatment with 2 μM Aminopurvalanol A had no detectable effects (63.4 ± 8.7% fertilized oocytes, 84.9 ± 7.1% of polyspermic oocytes, and 4 spermatozoa/polyspermic oocyte; *p* > 0.05 vs. CTRL), while at 10 μM it exerted a statistically significant effects (*p* < 0.05 vs. CTRL and 2 μM) in term of percentage of fertilized oocytes (45.5 ± 4.5% fertilized oocytes, 83.6 ± 4.9% of polyspermic oocytes and 4.3 spermatozoa/polyspermic oocyte). At 20 μM we did not found fertilized oocytes.

**Figure 7:**
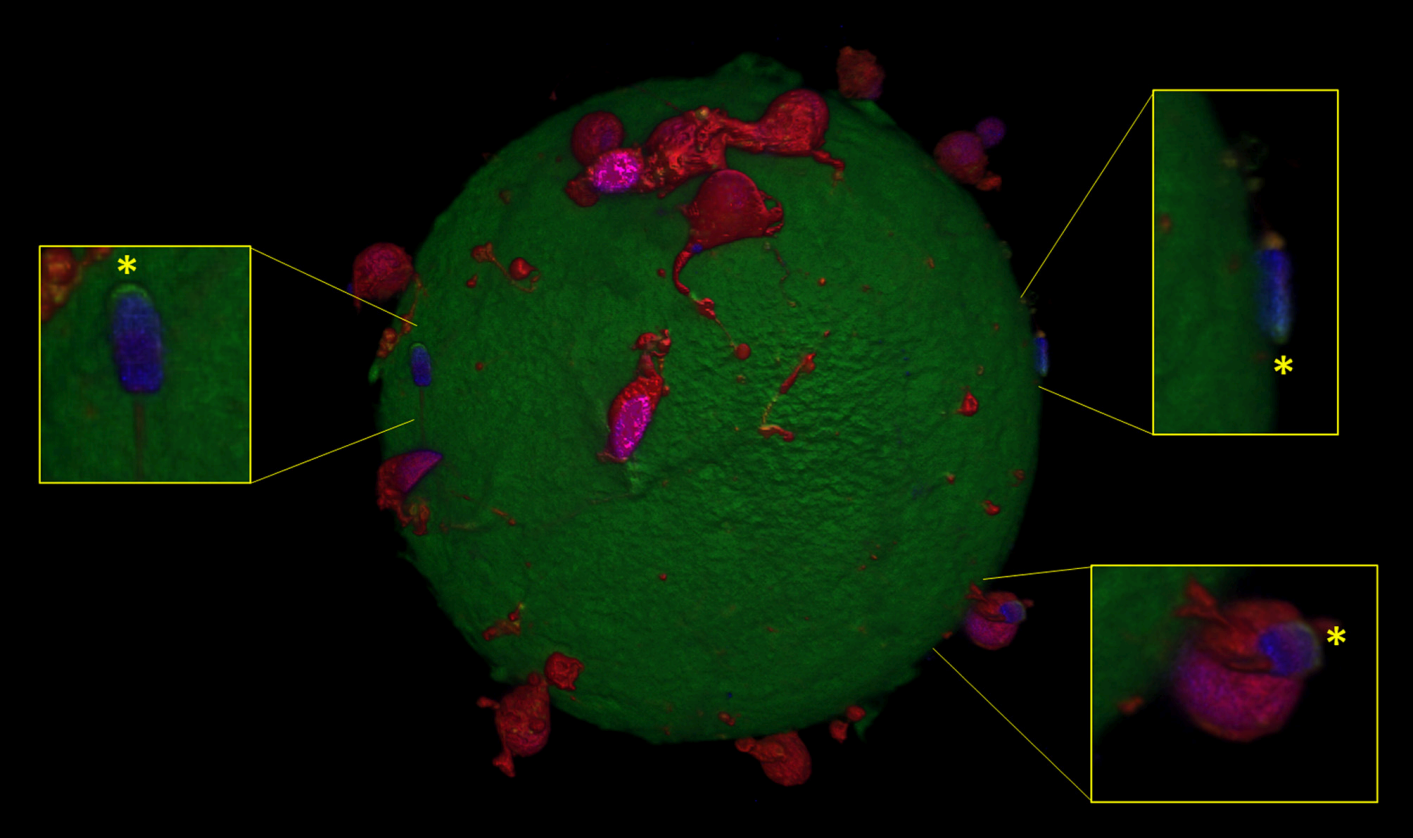
Confocal microscopy image showing spermatozoa with intact spermatozoa (*) adherent to oocyte zona pellucida, in presence of Aminopurvalanol A.

Interestingly, we found also that Aminopurvalanol A treatment was able to interfere with the ability to exocytate the acrosome in spermatozoa adherent to zona pellucida. Indeed, under control condition and with Aminopurvalanol A 2 μM we found that more than 60% of spermatozoa (61/92 and 51/82, respectively) showed absent or damaged acrosomes, while at 10 μM this percentage was reduced at about 30% (25/76) and at 20 μM only less than the 10% of spermatozoa were reacted (6/73)(see Figure 7).

### Effect of aminopurvalanol a on Erk1/2 activation

**Figure 8:**
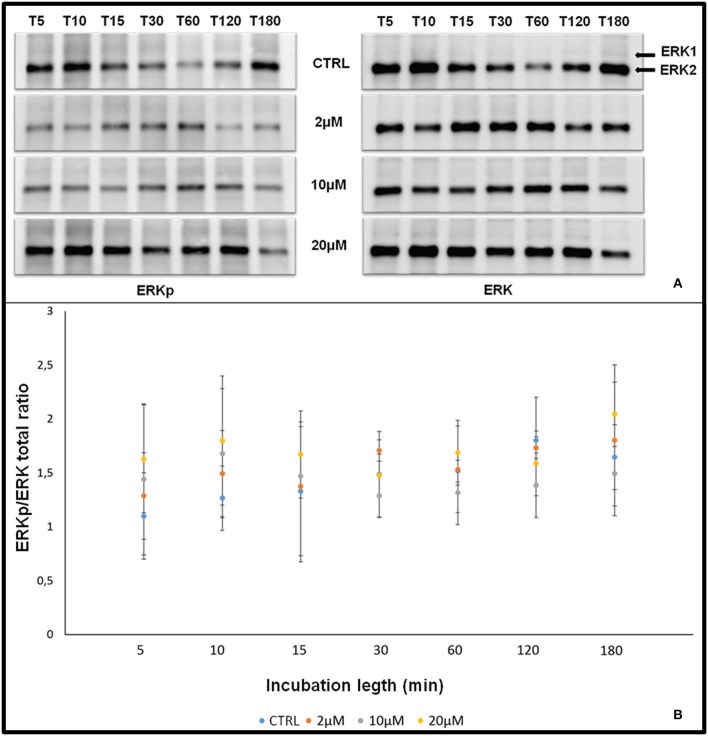
Effect of Aminopurvalanol A treatment on P-Erk/total Erk ratios. Graph showing the effect of of different times of incubation [5 min (T5), 10 min (T10), 15 min (T15), 30 min (T30), 1 h (T1h), 2 h (T2h), 3 h (T3h)] and Aminopurvalanol A concentration on P-Erk/total Erk ratios. The significance of the differences was determined by one-way ANOVA (*P* > 0.05 for all P-Erk/total Erk ratios vs. CTRL).

To exclude any effect of Aminopurvalanol A treatment on Erk1/2 activation we assessed the P-Erk/total Erk ratios. As it is shown in Figures 8A,B we did not find any relevant effect of the treatment on this parameter.

## Discussion

In recent years, some data converge in suggesting the presence of proteins involved in cell cycle and in cell cycle control pathways in mature spermatozoa (Amaral et al., 2014). Until now, there are not reliable hypothesis on their possible function in this context, indeed, it is known that sperm cells did not undergo cell cycle and that they are virtually transcriptionally silent.

To explore this intriguing enigma we realized an *in silico* and *in vitro* experiment. First of all, we used an approach based on the biological networks theory to build a model of proteins involved in cell cycle, with the purpose to identify the most influencing ones. Then, we assessed their function *in vitro* by using a specific inhibitor.

The network we obtained (we refer our analysis to CCN_MC, the main connected component of CCN, containing the 99.4% of nodes), is highly clustered (clustering coefficient = 0.880) and has an ultra-small world topology (characteristic path length = 2.397). This specific pattern is very interesting and differs for substantial aspect from the already known models of random or scale free (either following the BA model or hierarchical). In our opinion, this topology offers important advantages related to the control of cell cycle, which is one of the most delicate biological mechanisms. In particular, it assures the stability of the function thanks to the extremely high redundancy and the number of molecules interacting each other (in average each molecule is directly connected with over 62 other molecules). We used a multi-parametric approach to identify the nodes exerting the stronger control within CCN_MC. In detail, we performed a cluster analysis, based on different parameters related to the number of interactions and to the centrality of each node: node degree, betweenness centrality, closeness centrality, and stress centrality. Its results suggest that the node “CDK1”, corresponding to cdk1 has a peculiar role and that it exerts the higher control in CCN_MC. Noteworthy, CDK1, CCNB1, and CCNA2 are also the higher score bottleneck in the network.

Clearly, from a biological point of view, we consider cdk1 as an element of cyclin/cdk complex, and it is suggestive that also the nodes corresponding to the Cyclins play a key role in control on CCN_MC, as suggested by cluster analysis.

Despite it is well-studied the role of cyclin/cdk complexes in spermatogenesis, where they are involved in control of cell growth, differentiation, and apoptosis (Clement et al., 2015; Panigrahi et al., 2017; Wang et al., 2017), it is surprising that it could be possible to hypothesize an active role of these molecules in capacitation. To our knowledge, so far, there is a limited number of studies on their possible involvement in physiology of mature male gametes. In an old paper (Naz et al., 1993), it was proposed that cyclins and cdc2 serine/threonine protein kinase could be involved in human sperm function. Indeed, those authors found an immunopositivity for cyclin A and cyclin B1in the acrosomal regions of human spermatozoa and identified the specific band of p60 (cyclin A) and p62 (cyclin B1) on the Western blot. In both cases, the antibodies reacted more strongly with the specific cell region/band of capacitated sperm than with that of non-capacitated sperm. More interestingly, the cyclin A antibodies (but not the cyclin B1 antibodies) and cdc2 antibodies as well as the PSTAIRE antibodies significantly (*p* = 0.02 to *p* < 0.001) increased the human sperm penetration rates of zona-free hamster ova; the cyclin A and cdc2 antibodies showed the strongest enhancing effects. These three antibodies significantly increased the acrosome reaction and release of acrosin activity from the sperm cells.

Several years after, it was found that the incubation of dog spermatozoa with 10 mM glucose increased serine phosphorylation of cyclins B and E, Cdk2, Cdk6, Cdc6, PYK2, c-kit, Raf-1, TRK, and several other protein phosphatases related to cell cycle control (Fernández-Novell et al., 2011). Furthermore, the incubation of dog spermatozoa with 10 mM fructose decreased serine phosphorylation levels of cyclins B and D3, Cdk1/Cdc2, Cdk2, Cdk6, Akt, PI3 kinase, ERK-1, and protein kinase C. On the other hand, the incubation of boar spermatozoa with these monosaccharides did not modified the phosphorylation patterns studied. The Authors concluded that these data suggest that these molecules are not only expressed by male gametes, but they are also active in controlling their function.

More recently the phosphorylation of CDK1/CDC2 has also been observed after liquid-storage of boar semen at 17°C (Yeste et al., 2014).

These data are surprising, because the cyclin/cdk system is known to be strictly involved in control of cell cycle. Indeed, it has been found to be present in all known eukaryotes where it acts as a regulatory factor of cell cycle. In animal cells at least nine CDKs have been described, four of which (CDK1, 2, 3, and 4) are directly involved in cell cycle control (Rev et al., 1997). For each phase of cells cycle a specific cyclin/cdk has been demonstrated to be active as controller. For instance, cyclin c/Cdk3 are involved in G0 phase, cyclin D and E and Cdk 4, 2, and 6 (respectively) in G1, Cyclin A and E and Cdk2 in S, cyclin A and Cdk 2 and 1 in G2, and Cylin B and Cdk2 in M (Satyanarayana and Kaldis, 2009).

To investigate the potential role of these molecules, coherently with the approach used, before to carry out *in vitro* experiments, we simulated *in silico* the effect of a potent and specific cell permeable inhibitor of the formation of Cyclin/Cdk complex, that Aminopurvalanol A inhibits (Knockaert et al., 2000). Considering that in literature and on the supplier's site, it has been indicated that inhibits specifically the activity of cdk1/cyclin B, cdk2/cyclin A, cdk5/p25 and cdk4/cyclin D complexes, we removed from CCN_MC the following nodes: cdk1, cdk2, cdk4, cdk 5, cyclin A, cyclin B, cyclin D, and p25 (see Figure 1B). Since we found significant changes in network topology, we switched to the *in vitro* model. To our knowledge, Aminopurvalanol A has been never used in spermatozoa, thus as first we identified the dose able to promote a biologically relevant effect without inducing cytotoxicity. Based on the result of the dose-effect curve we realized we decided to use different concentrations (2, 10, 20 μM). These value are partially in keeping with those reported in other cellular model in which it has been shown that it is able to arrest cell cycle at G_2_/M boundary with a IC_50_ = 1.25 μM. Some difference could be justified by several unpredictable factors, such as cell permeability, intracellular metabolism of the compound, competition with high intracellular concentration of ATP, and interaction with other targets, that could interfere with its activity (Knockaert et al., 2002b).

After we have identified the Aminopurvalanol A concentrations able to inducing detectable biological effect without being toxic, we studied their effects on sperm capacitation-related events. In somatic cells, the specific inhibitory effect of Aminopurvalanol A is evaluated assessing the induction of cell cycle arrest at G_2_/M phase, but obviously this parameter in not measurable in spermatozoa we considered the integrity of acrosomes, which is strictly related with the fertility.

It was immediately evident that the drug promotes the loss and/or the damage of acrosomes during the incubation under capacitating conditions. This effect is dose and time dependent and is negligible for 2 μM dose (*p* > 0.05 vs. CTRL), while become to be detectable at 2 or 4 h in 20 and 10 μM doses, respectively (*p* < 0.05 vs. CTRL). The effects of 10 and 20 doses are different in terms of E50, and spermatozoa treated with the higher concentration of Aminopurvalanol A display an early damage. It is very interesting that we found an early and important effect of the drug on acrosome integrity at a concentration of 20 μM, besides it has been found that in different cellular models Aminopurvalanol A induces apoptosis at concentrations >10 μM (Knockaert et al., 2002a). In this respect, we can hypothesize that a similar effect could be maintained in part. Indeed the membrane remodeling and the onset of membrane instability in spermatozoa is closely related both with capacitation and with apoptosis.

In physiological conditions, during capacitation, the globular actin (G-actin) polymerizes forming long filaments of F-actin that form a network acting as a diaphragm between outer acrosome membrane (OAM) and plasma membrane (PM). When the capacitation progresses these structures become more fusogenic and the interposition of F-actin avoids their premature fusion. These events are the result of the activation of a complex pathway involving cAMP/PKA, RhoA/C and Rac1, that leads the phosphorylation of LIM Motif-Containing Protein Kinase 1 (LIMK1), that in turn promotes the transient phosphorylation of Cofilin, which no longer binds and severs F-actin (Romarowski et al., 2015). Only when the physiological stimulus (the proteins of oocyte ZP) is met, a fast peak of intracellular concentration of calcium allows the depolymerization of actin and ultimately the PM and OAM contact and fusion (Bernabò et al., 2010b; Daniel et al., 2010). Therefore, the insufficient actin polymerization could lead to the loss of acrosome integrity (Shabtay). This is the reason why we assessed the actin polymerization in spermatozoa treated with Aminopurvalanol A. Epifluorescence studies clearly demonstrated as the capacitation-dependent increase in actin polymerization was antagonized by the Aminopurvalanol A in a dose and time dependent manner. As reported in Figure 2, while in CTRL samples the incubation under capacitating conditions promotes the increase in the percentage of sperm cells showing higher levels of fluorescence, in 10 and, more, in 20 μM treated samples this event is markedly reduced. Interestingly, the single cell confocal scanning confirmed this datum and showed that this event markedly involved the anterior region of sperm head, where actin avoids the premature fusion of OAM and PM (Figure 4).

We examined also the Aminopurvalanol A effect on tubulin cytoskeleton, finding that it did not exert any detectable effect on this parameter. For the same reason, in parallel, we evaluated the effect of the drug on capacitation-dependent membrane remodeling by assessing the Calculated Diffusion Coefficient of a fluorescent probe, Dil C12 (Gadella and Harrison, 2000), we measured the. The results demonstrated that Aminopurvalanol A did not exert any detectable effect on membrane fluidity in intact spermatozoa, leading us to conclude that the effects of the drug we have demonstrated are not due to a detrimental effect of AP on sperm physiology, but are specifically due to the effect on actin cytoskeleton organization.

Since in other cellular model it has been reported that higher doses of Aminopurvalanol A could interfere with MAPK pathways, we assessed the P-Erk/total Erk. We found that it is unaffected by the concentrations of Aminopurvalanol A that we used in our experiments, thus allowing us to affirm that the effects we documented are actually related to the activity of cyclin/cdk complexes.

Ultimately, to assess the impact in term of fertilizing ability of the alteration induce by Aminopurvalanol A, we carried out an IVF experiment with spermatozoa incubated for 2 h under capacitating conditions, with the aim of verify if the effect on acrosome integrity could be able to interfere with sperm fertilizing ability.

Interestingly, we found that the acrosome damage was paralleled by statistically significant (*p* < 0.01) reduction of fertilizing ability of treated spermatozoa. To investigate this last point, we checked the effect of Aminopurvalanol A on the number of fertilized oocytes, the number of polyspermic oocytes/fertilized oocyte, and the number of spermatozoa/polyspermic oocyte after IVF, because particularly in swine model (Abeydeera and Day, 1997; Suzuki et al., 2003) these parameters are related with the capacitation status and fertility of semen (Hunter and Nichol, 1988; Abeydeera et al., 1997). We found that all the parameters related to the fertilizing ability were negatively affected (*p* < 0.05, in comparison with CTRL samples) by the treatment. This finding, in our opinion, suggests that the spermatozoa that lost the acrosome integrity are those undergoing capacitation.

These results suggest that in mature spermatozoa cyclin/cdk complexes could maintain an important role in controlling the actin polymerization during capacitation. It is possible to hypothesize that cyclin/cdk complexes could be involved in activating/stimulating/maintaining of actin polymerization, and that the treatment with Aminopurvalanol A could negatively interfere with that process. This hypothesis is strengthened by the reduced fertilizing ability of sperm samples treated with Aminopurvalanol A documented by IVF experiments. Interestingly, in treated samples we found a very lower number of spermatozoa adherent to the ZP or embedded in cumulus with exocytated acrosomes, thus confirming the reduction of percentage of capacitated cells.

In conclusion, here we reach two different important results:

1. We demonstrated the utility of computational modeling strategies in exploring cell signaling systems. The adoption of a biological network-based approach allowed us to infer new and interesting processes involved in sperm capacitation and the simulation we carried out was confirmed by the experimental results;
2. We documented the effect of Aminopurvalanol A, a potent and selective permeable inhibitor of cyclin/cdk complexes formation, on rearrangement of actin cytoskeleton of spermatozoa during capacitation. We cannot exclude indirect or other effects of this drug, but altogether these data seem to suggest a new element in control system of actin polymerization during boar sperm capacitation. Since cyclin/cdk complexes are highly conserved (Malumbres, 2014), we can easily hypothesize that this mechanism could be shared by the other mammalian species.

In our opinion, this finding could be of great interest for different reasons. On the one hand, it complete the knowledge about the non-canonical functions of cell cycle cyclins and cyclin-dependent kinases (see Hydbring for a review of this topic, Hydbring et al., 2016). On the other hand, it potentially opens the way for further studies on the physiology and pathology of sperm capacitation.

## Author contributions

NB conceived the study and drafted the manuscript; LV realized the confocal microscopy experiments; LG, GC, and MR, realized immunocytochemistry and IVF experiments; PP and LB realized the biochemistry experiments; BB and MM revised the manuscript. All were involved in acquisition and interpretation of data, and in approval of the final version of the manuscript.

### Conflict of interest statement

The authors declare that the research was conducted in the absence of any commercial or financial relationships that could be construed as a potential conflict of interest.
