# Supplementary figures and images for: Aminopurvalanol A, a Potent, Selective, and Cell Permeable Inhibitor of Cyclins/Cdk Complexes, Causes the Reduction of in Vitro Fertilizing Ability of Boar Spermatozoa, by Negatively Affecting the Capacitation-Dependent Actin Polymerization
Source: Front Physiol. 2017 Dec 22;8:1097. doi: 10.3389/fphys.2017.01097 (PMC5744433; doi:10.3389/fphys.2017.01097)

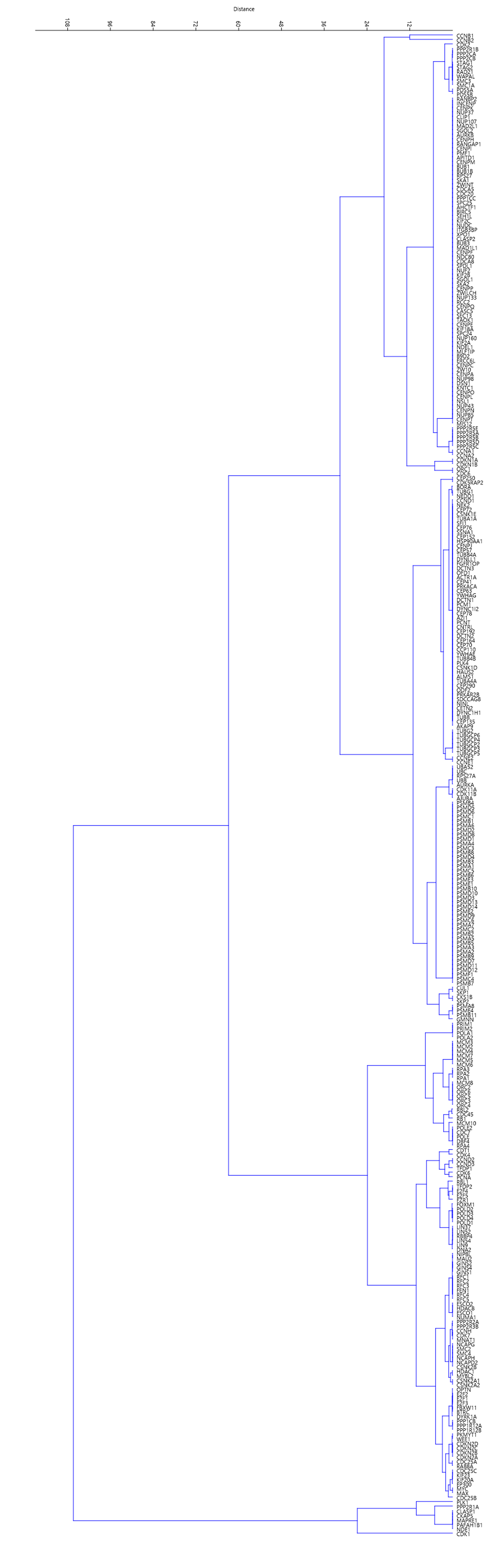

Supplement: Supplementary file 1 [file Image1.tif]
